# Supplementary figures and images for: Genome-Wide Identification of the PP2C Gene Family and Analyses with Their Expression Profiling in Response to Cold Stress in Wild Sugarcane
Source: Plants (Basel). 2023 Jun 22;12(13):2418. doi: 10.3390/plants12132418 (PMC10346257; doi:10.3390/plants12132418)

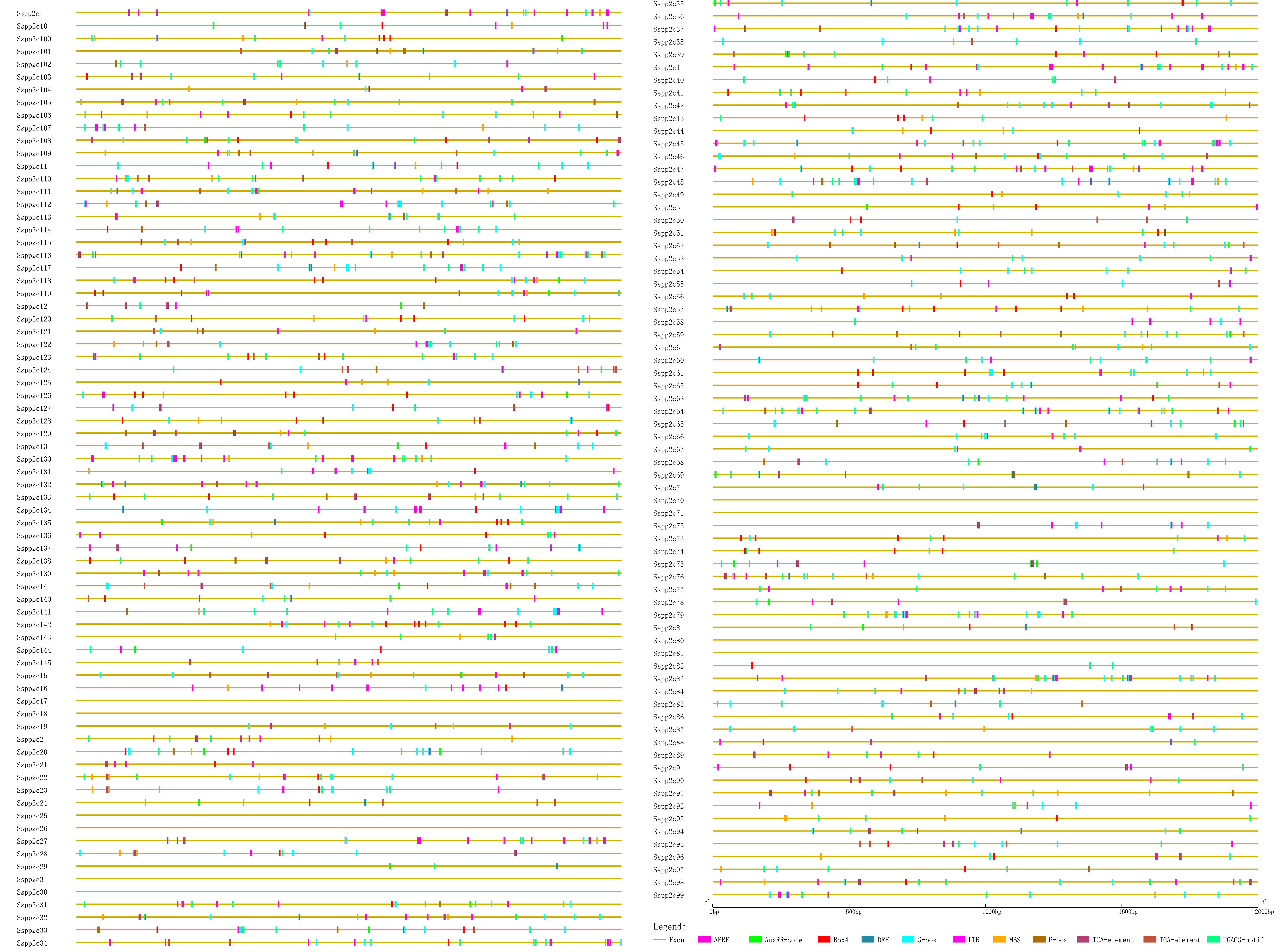

Supplement: Supplementary file 1 [file plants-12-02418-s001.zip › Figure S2.tif]

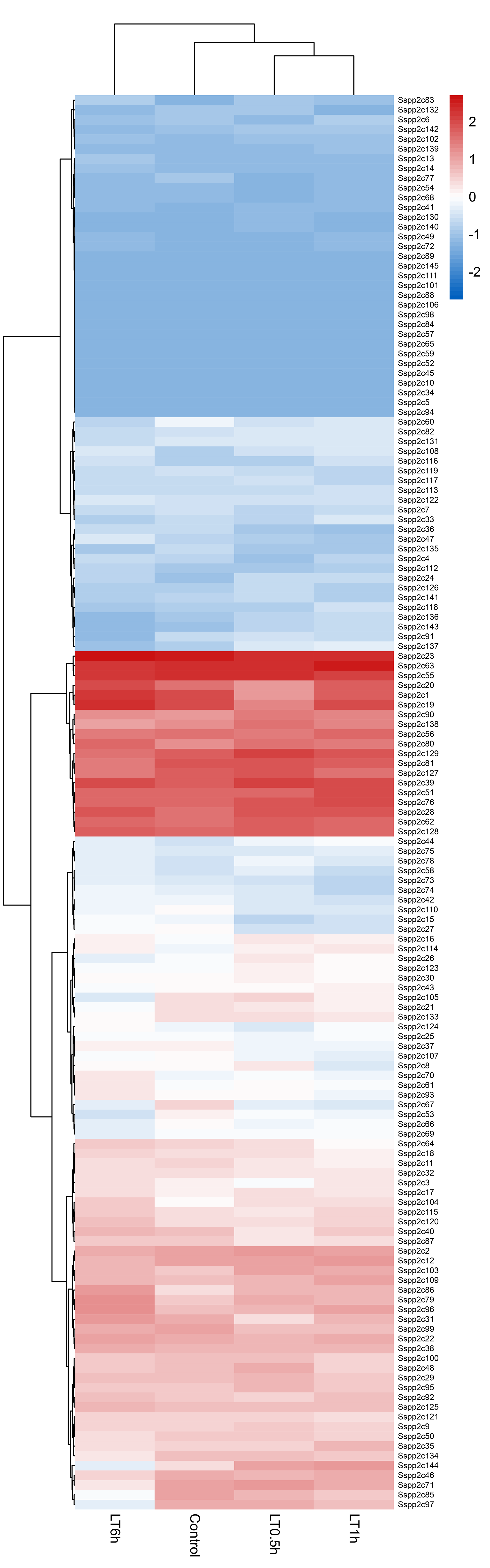

Supplement: Supplementary file 1 [file plants-12-02418-s001.zip › Figure S3.tif]

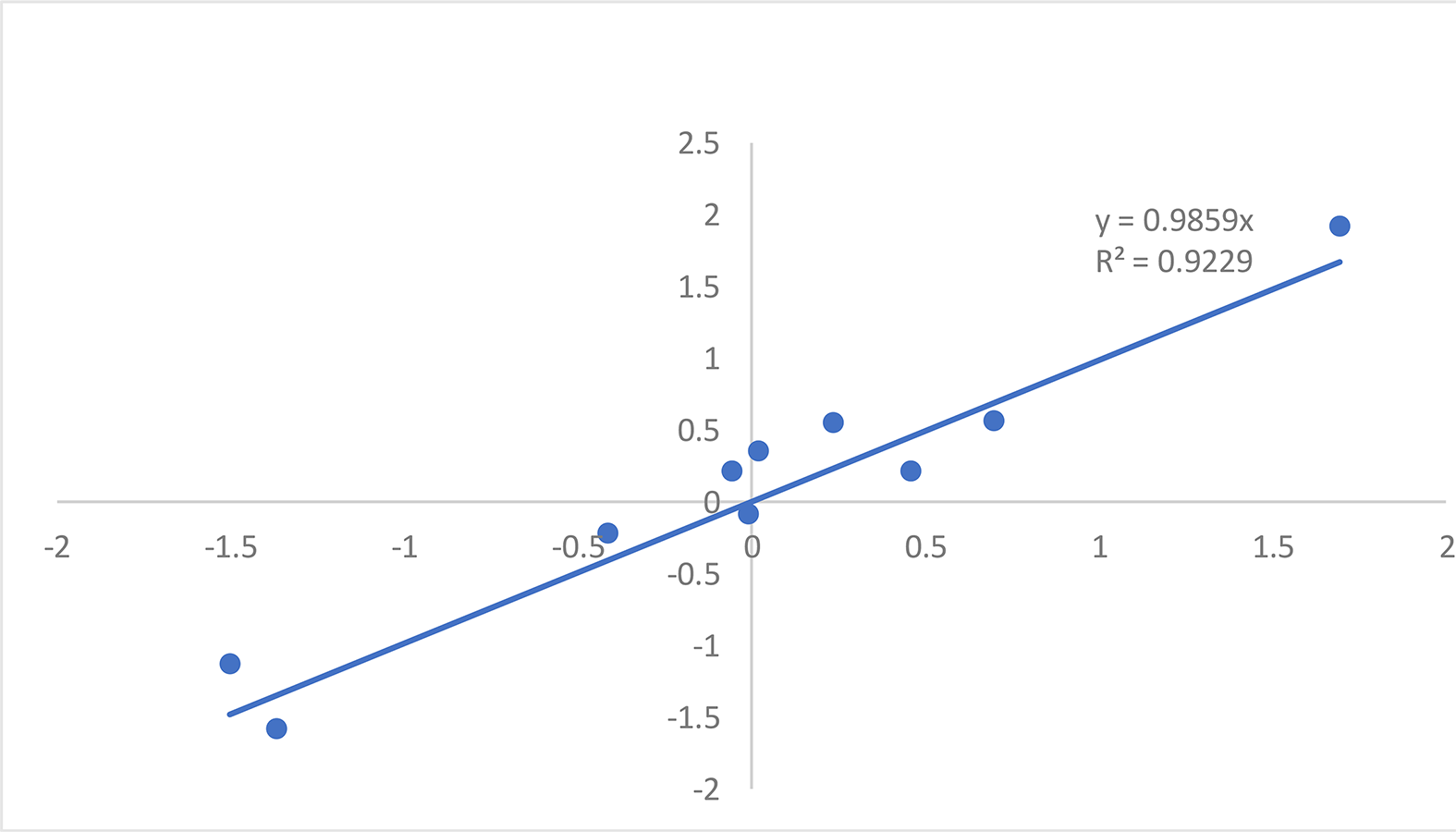

Supplement: Supplementary file 1 [file plants-12-02418-s001.zip › Figure S4.tiff]

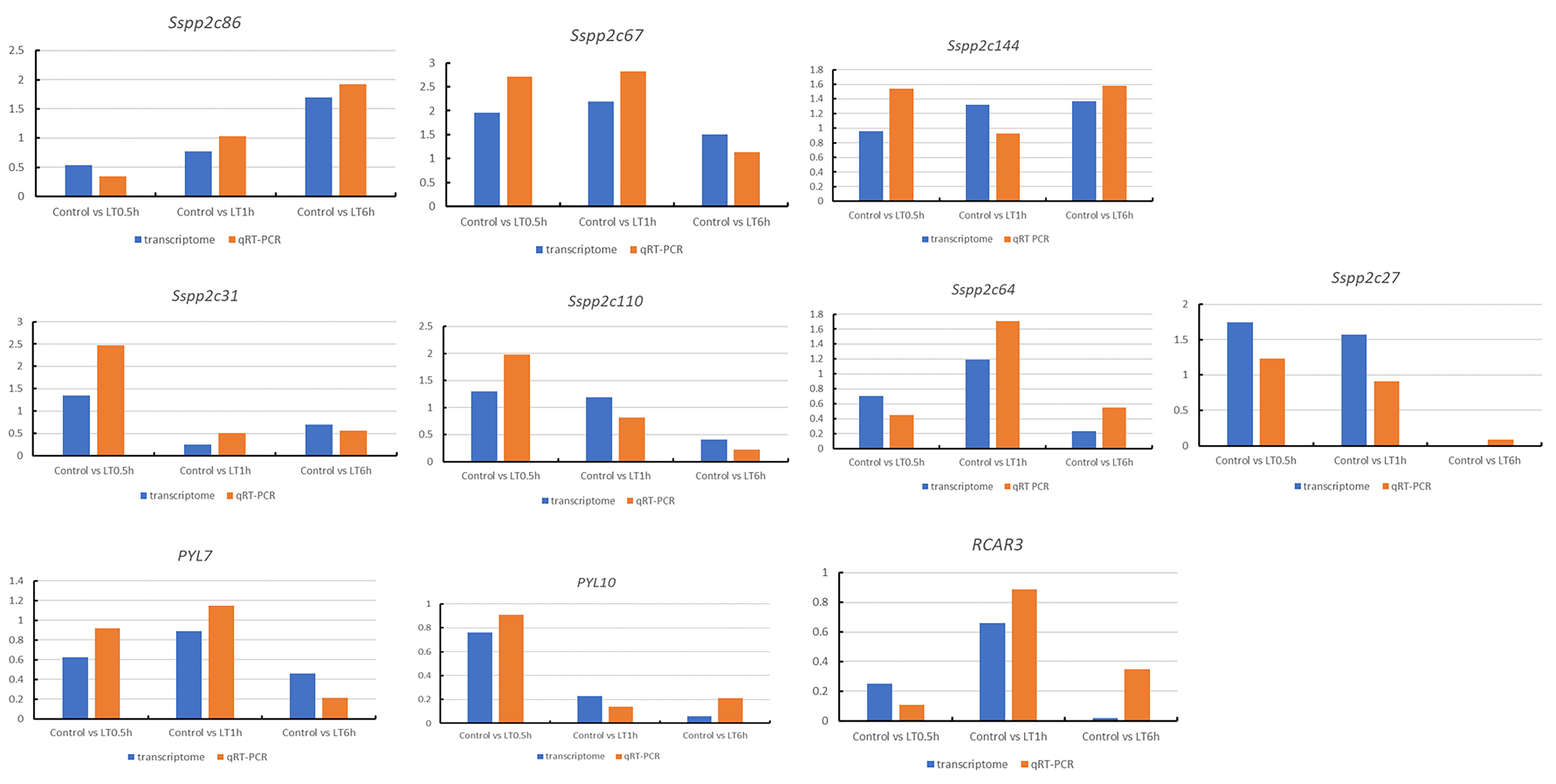

Supplement: Supplementary file 1 [file plants-12-02418-s001.zip › Figure S5.tif]
